# Highly Efficient Genome Editing in Plant Protoplasts by Ribonucleoprotein Delivery of CRISPR-Cas12a Nucleases

**DOI:** 10.3389/fgeed.2022.780238

**Published:** 2022-01-31

**Authors:** Yingxiao Zhang, Yanhao Cheng, Hong Fang, Nathaniel Roberts, Liyang Zhang, Christopher A. Vakulskas, Randall P. Niedz, James N. Culver, Yiping Qi

**Affiliations:** ^1^ Department of Plant Science and Landscape Architecture, University of Maryland, College Park, College Park, MD, United States; ^2^ College of Agriculture, Nanjing Agricultural University, Nanjing, China; ^3^ Integrated DNA Technologies, Coralville, IA, United States; ^4^ U.S. Horticultural Research Laboratory, USDA-Agricultural Research Service, Fort Pierce, FL, United States; ^5^ Institute for Bioscience and Biotechnology Research, University of Maryland, Rockville, MD, United States

**Keywords:** CRISPR-Cas12a, ribonucleoprotein, genome editing, rice, citrus, protoplast

## Abstract

Clustered Regularly Interspaced Short Palindromic Repeats (CRISPR) mediated genome editing is a powerful approach for crop improvement. Traditional transformation methods based on plasmid delivery pose concerns associated with transgene integration and off-target effects. CRISPR delivered as ribonucleoproteins (RNPs) can prevent exogenous DNA integration, minimize off-target effects, and reduce cellular toxicity. Although RNP delivered CRISPR genome editing has been demonstrated in many plant species, optimization strategies that yield high editing efficiencies have not been thoroughly investigated. Using rice and citrus protoplast systems we demonstrated highly efficient genome editing using Cas12a delivered as RNPs. Four Cas12a variants, including LbCas12a, LbCas12a-E795L, AsCas12a, and AsCas12a Ultra, were investigated. Nearly 100% editing efficiency was observed for three out of four target sites by LbCas12a, LbCas12a-E795L, and AsCas12a Ultra, as measured by restriction fragment length polymorphism (RFLP) and verified by next generation sequencing of PCR amplicons. RNP delivery resulted in higher editing efficiencies than plasmid delivery at 32°C and 25°C. LbCas12a and LbCas12a-E795L demonstrated increased editing efficiencies in comparison to AsCas12a and AsCas12a Ultra, especially when used at lower RNP concentrations. In addition, we discovered that a 1:1 Cas12a:crRNA molar ratio is sufficient to achieve efficient genome editing. Nuclear localization signals (NLSs) are essential for efficient RNP-based genome editing. However, the different crRNA modifications tested did not significantly improve genome editing efficiency. Finally, we applied the Cas12a RNP system in citrus protoplasts and obtained similarly high editing efficiencies at the target site. Our study provides a comprehensive guideline for Cas12a-mediated genome editing using RNP delivery in plant cells, setting the foundation for the generation of transgene-free genome edited plants.

## Introduction

CRISPR-Cas12a (formerly Cpf1) is a Class II type V CRISPR system widely used for genome editing in plants (Zhang et al., 2019a; Hassan et al., 2021). Commonly used Cas12a nucleases in eukaryotes include LbCas12a and AsCas12a (Zetsche et al., 2015; Zhang et al., 2019a; Hassan et al., 2021). In plants, the most predominantly used Cas12a is LbCas12a, which has been demonstrated in *Arabidopsis* (Bernabe-Orts et al., 2019; Malzahn et al., 2019; Schindele and Puchta, 2019), rice (Begemann et al., 2017; Hu et al., 2017; Tang et al., 2017; Wang et al., 2017; Xu et al., 2017; Yin et al., 2017; Zhong et al., 2018; Li et al., 2019b), maize (Lee et al., 2018; Malzahn et al., 2019), *Nicotiana benthamiana* (Bernabe-Orts et al., 2019), tomato (Bernabe-Orts et al., 2019), lettuce (Kim et al., 2017), cotton (Li B. et al., 2019), citrus (Jia et al., 2019), and poplar (An et al., 2020). FnCas12a also displays efficient genome editing in plants (Endo et al., 2016; Begemann et al., 2017; Wang et al., 2017; Zhong et al., 2018). However, AsCas12a, despite its success in mammalian genome editing, has low nuclease activity in plants, which is likely due to its high sensitivity to low temperature (Tang et al., 2017; Malzahn et al., 2019). More recently, a suite of new Cas12a orthologs were reported for efficient genome editing in rice (Zhang et al., 2021c).

Compared to the most popular *Streptococcus pyogenes* Cas9 (SpCas9) system, Cas12a has multiple distinct features. First, Cas12a proteins recognize T-rich protospacer adjacent motifs (PAMs), while SpCas9 recognizes a G-rich NGG PAM. Although the canonical PAM for Cas12a is TTTV, the recently reported Mb2Cas12a recognizes a relaxed TTV PAM (Zhang et al., 2021c). Second, Cas12a uses a short CRISPR RNA (crRNA) for DNA targeting whereas Cas9 utilizes a crRNA and a tracrRNA or their combined single guide RNA (sgRNA) for DNA targeting. The use of a ∼43 nt short crRNA makes the Cas12a system suitable for multiplexed editing. It also reduces the cost for guide RNA synthesis when CRISPR reagents are delivered as ribonucleoproteins (RNPs). Third, Cas12a generates staggered ends, cutting at distal sites from the PAM, while Cas9 generates blunt end cutting at a site only 3 bp from the PAM. Hence, Cas12a is more likely to cut and edit the target site repeatedly, resulting in larger deletions (Zetsche et al., 2015; Tang et al., 2017) or enhanced homology-directed repair (HDR) (Li et al., 2019c; Wolter and Puchta, 2019). Fourth, Cas12a also possesses nuclease activity to cleave RNA, resulting in self-processing of the crRNA repeats, which further aids multiplexed editing as demonstrated in human cells (Zetsche et al., 2017), and plants (Wang et al., 2017). Finally, Cas12a appears to be more specific than Cas9, resulting in less off-targeting potential (Kim et al., 2016; Kleinstiver et al., 2016; Tang et al., 2018).

While these above-mentioned features make Cas12a advantageous to Cas9, there are two major limitations of Cas12a. One reason is Cas12a has reduced nuclease activity at lower temperatures that are relevant for genome editing in plants (Malzahn et al., 2019). High temperature regimes were applied to improve Cas12a genome editing in many plant species such as *Arabidopsis*, rice and maize (Malzahn et al., 2019). Alternatively, engineered Cas12a variants such as LbCas12a-D156R (Kleinstiver et al., 2019) was applied for improved genome editing in *Arabidopsis* (Schindele and Puchta, 2019) and *Drosophila* (Port et al., 2020). The second reason is unlike Cas9, Cas12a nickases have not been engineered, which largely prevents its use in base editing and prime editing. Efficient base and prime editors all use Cas9 nickases that cut only one strand of DNA (Komor et al., 2016; Gaudelli et al., 2017; Anzalone et al., 2019). Cas12a base editors constructed with deactivated Cas12a (dCas12a) were developed for genome editing in human cells with low efficiency (Li et al., 2018; Kleinstiver et al., 2019), discouraging their applications in plants. It is believed that development of efficient reagent delivery systems could partly overcome these limitations, thus expanding Cas12a genome editing capability in plants. RNP delivery of CRISPR-Cas systems is a promising approach for genome editing in plants (Zhang et al., 2021b). Although genome editing in plants has traditionally relied on DNA delivery methods based on *Agrobacterium*, gene gun, and or virus, RNP delivery has multiple advantages over these methods. First, RNP delivery represents a transgene- or virus-free method that can mitigate the potential regulatory problems in genome-edited crops. Second, RNP delivery bypasses the cellular transcription and translation processes, which can enhance cell targeting, and increasing genome editing efficiency. Third, RNP delivery is transient, which will reduce potential off-target effects. Fourth, RNP delivery could enable controllable genome editing efficiencies through the use of different RNP dosages. Fifth, RNP delivery allows for convenient multiplexed genome editing as multiple crRNAs can be complexed with Cas12a for simultaneous delivery. Finally, RNP delivery could benefit from potential protein and/or RNA modifications to produce tunable genome editing outcomes that otherwise are nearly impossible to implement through DNA/virus-based delivery. Plant protoplasts is a great platform for assessing genome editing reagents (Yoo et al., 2007; Lin et al., 2018), especially through RNP delivery (Kim et al., 2017; Andersson et al., 2018; Zhang et al., 2021b). Therefore, we wanted to assess RNP delivery of CRISPR-Cas12a reagents using protoplast systems of rice (a monocot) and citrus (a dicot), which allowed us to test different parameters to optimize the CRISPR-Cas12a delivery regime that can be widely applied in many plant species.

## Materials and Methods

### Plant Materials

Rice (*Oryza sativa*) Japonica cultivar Nipponbare were grown on ½ MS basal salt medium (Murashige and Skoog, 1962) in the dark at 28°C for 14 days. Etiolated rice leaves were used for protoplast isolation. Suspension cells derived from embryogenic calli of Hamlin sweet orange (*Citrus sinensis*) line H89 were maintained in liquid MT (Murashige and Tucker) media (Murashige and Tucker, 1969) (Phytotech Catalog #M5525) and subcultured every 3 weeks.

### Cas12a and crRNA Reagents and RNP Assembly

All Cas12a variants with or without nuclear localization signals (NLSs) (Behlke et al., 2018; Vakulskas et al., 2020; Zhang et al., 2020) and crRNAs with or without end modifications were provided by IDT (Integrated DNA Technologies, Inc., United States). LbCas12a crRNA scaffold sequence was used for LbCas12a genome editing, while AsCas12a crRNA scaffold sequence was used for AsCas12a. All crRNA sequences are included in Supplementary Table S1. To assemble RNP to a final concentration of 0.1 µM in the protoplast cell culture, with a 1:5 Cas12a:crRNA ratio, 20 µg Cas12a nuclease was mixed with 667 pmol crRNA in a 1 X NEBuffer™ 3.1 solution. Reduced amount of Cas12a nuclease and crRNA with the same ratio were applied to assemble RNP to lower final concentrations. The 20 µl mixture was then incubated at room temperature for 10 min. The amount of Cas12a and crRNA used for each protoplast transfection was adjusted according to the concentration and Cas12a:crRNA ratio. The 1:5 Cas12a:crRNA ratio was used for rice protoplast transfection except when testing different ratios. The 1:2 Cas12a:crRNA ratio was used for citrus protoplast transfection to ensure enough crRNAs were provided.

### Vector Construction

T-DNA vectors were constructed as previously described (Tang et al., 2017; Zhang et al., 2019b). The crRNA targeting *OsEPFL9* was synthesized as duplexed oligonucleotides, which were phosphorylated, annealed, and ligated into pYPQ141-ZmUbi-RZ-As (for AsCas12a-Ultra, Addgene #86196), and pYPQ141-ZmUbi-RZ-Lb (for LbCas12a and LbCas12a-E795L, Addgene #86197) at the BsmBI site (Supplementary Table S2) (Tang et al., 2017). Mutations were introduced to pYPQ230 (Addgene #86210) and pYPQ220 (Addgene #86208) (Tang et al., 2017) to generate LbCas12a-E795L expression vector pYPQ230-E795L (Addgene #176890) and AsCas12a Ultra expression vector pYPQ220-Ultra (Addgene #176889), respectively, and using the Q5® Site-Directed Mutagenesis Kit (New England BioLabs). These three Cas12a expression vectors (pYPQ230, pYPQ230-E795L, and pYPQ220-Ultra) were assembled with their corresponding crRNA expression vectors and the destination vector pYPQ203 (Addgene #86207) (Tang et al., 2017) using the three-way Gateway assembly (Zhang et al., 2019b).

### Rice Protoplast Transfection

Rice protoplasts were isolated and transfected as previously described (Lowder et al., 2015). For RNP delivery, 20 µl assembled RNP mixture (with different concentrations and/or Cas12a:crRNA ratios) was mixed with 200 µl protoplast (1 × 10^6^ cells/ml) and 220 µl PEG solution (40% (w/v) PEG 4000, 0.2 M mannitol, 0.1 M CaCl_2_). For plasmid delivery, 30 µl plasmid DNA (30 µg) was mixed with 200 µl protoplast (1 × 10^6^ cells/ml) and 230 µl PEG solution. After 30 min incubation at room temperature, the transfection was terminated by adding 900 μl W5 buffer. Protoplasts were collected by centrifugation and resuspended in 1.33 ml W5 buffer. The mixtures were transferred into 12-well culture plates and incubated at 32°C (the default temperature) or 25°C (only for Figure 2) in the dark for 2 days. The protoplasts were collected and lysed for target site amplification using the Phire Plant Direct PCR Kit (Thermo Scientific™).

### Citrus Protoplast Transfection

Suspension citrus cells derived from embryogenic calli of Hamlin 89 (H89) sweet orange were used for protoplast isolation and the following RNP delivery. To obtain suspension citrus cell culture, approximately 5 g of citrus embryogenic calli maintained on solid MT50 medium (MT (Murashige and Tucker, 1969) (Phytotech Catalog #M5525) (supplemented with 50 g/L sucrose, 50 µM 6-Benzylaminopurine and 8 g/L agar at pH 5.8) were crushed using a spatula and resuspended in 50 ml liquid MT50 media (MT50 medium without any agar). Suspended cells were maintained in liquid MT50 media in the dark at room temperature on an orbital shaker (120 rpm) and subsequently subcultured every 3 weeks. The cells were drained and digested in a filter-sterilized enzyme solution (0.6 M mannitol, 10 mM CaCl_2_, 10 mM MES buffer, and 0.75% (w/v) cellulase Onozuka RS (Yakult Pharmaceutical IND. CO., Tokyo, Japan), 0.75% (w/v) Macerozyme R-10 (Yakult Pharmaceutical IND. CO., Tokyo, Japan), 0.1% BSA, and pH 5.6) in the dark at room temperature on an orbital shaker (80 rpm) for approximately 16 h. The digested protoplasts were purified and transfected using the same method as for rice protoplasts, except that the transfected protoplasts were incubated at 28°C in the dark for 2 days. For RNP delivery in citrus, LbCas12a were used and different concentrations (0.1, 0.01, and 0.001 µM) of RNP were tested. Protoplasts transfected with water were used as the mock control. The protoplasts were collected and lysed for target site amplification using the Phire Plant Direct PCR Kit (Thermo Scientific™).

### Editing Efficiency Analysis by RFLP and Next Generation Sequencing

To measure editing efficiencies of RNP-mediated and plasmid-mediated targeted mutagenesis, the restriction fragment length polymorphism (RFLP) method was first used. Amplicons harboring target sites were digested and ran through 2% TAE agarose gels. Amplicons with edits are expected to lose restriction enzyme cutting sites, resulting in digestion-resistant bands. Editing efficiencies were quantified using ImageJ (https://imagej.nih.gov/ij/). Editing efficiency = undigested band intensity/(undigested band intensity + digested band intensity) × 100%. To measure editing efficiencies using amplicon deep sequencing, target sites were PCR amplified using the Phire Plant Direct PCR Kit (Thermo Scientific™). Each sample was barcoded and sequenced using the Illumina sequencing platform. Demultiplexed NGS data was analyzed for genome editing with CRISPAltRations v1.0.0 using default parameters and the recommended window size (9 bp) for detecting Cas12a editing (Kurgan et al., 2020). All primers used for RFLP assay and amplicon deep sequencing are listed in Supplementary Table S2.

### Statistical Analysis

Pairwise comparisons were conducted using Student’s t-test. Significant differences were indicated using one asterisk (*p* < 0.05), two asterisks (*p* < 0.01) or three asterisks (*p* < 0.001). Multiple comparisons are conducted using Tukey’s Honest Significant Difference (HSD) test in R. Treatments with the same letter are not significantly different when α = 0.05.

## Results and Discission

### Engineered Cas12a Variants Show Enhanced Activities in Rice Cells With RNP Delivery

**FIGURE 1:**
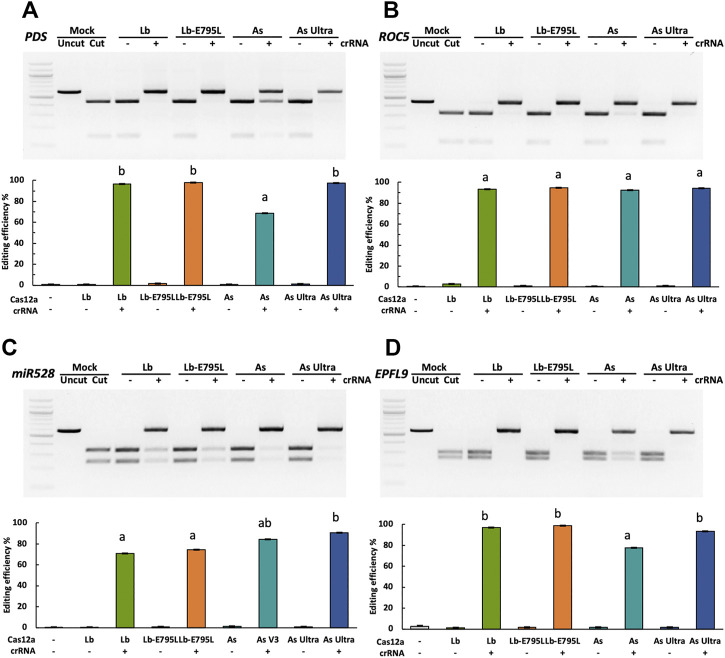
Engineered Cas12a variants show enhanced activities in rice cells with RNP delivery. Editing efficiencies of four Cas12a variants in rice cells with RNP delivery at the *OsPDS* **(A)**, *OsROC5* **(B)**, *OsmiR528* **(C)**, and *OsEPFL9* **(D)** sites at 32°C. Editing efficiencies are calculated using the RFLP assay. Data are presented as mean values ±SEM of three biologically independent replicates. Editing efficiencies of four Cas12a variants at each target site are compared using Tukey’s Honest Significant Difference (HSD) test. Treatments with the same letter are not significantly different when α = 0.05.

To investigate whether engineered Cas12a variants can produce higher editing efficiencies, four Cas12a variants, including LbCas12a, LbCas12a-E795L, AsCas12a, and AsCas12a Ultra (Supplementary Table S3) were used to target four genes (*OsPDS*, *OsROC5*, *OsmiR528,* and *OsEPFL9*) in rice protoplasts individually. Cas12a and its corresponding crRNAs were delivered as RNPs. Genome editing efficiencies were evaluated using the RFLP assay (Figure 1). When Cas12a nucleases were delivered without crRNAs, no editing activity was observed. RNP delivery resulted in highly efficient genome editing at all four target sites. At the *OsPDS* and *OsEPFL9* sites, LbCas12a, LbCas12a-E795L, and AsCas12a Ultra showed high editing efficiencies, 93.6–98.8%, which outperformed AsCas12a at 68.5–77.6% (Figures 1A,D). At the *OsROC5* site, 92.5–94.6% editing efficiencies were obtained with all four variants, showing no significant differences among these variants (Figure 1B). Slightly lower editing efficiencies were observed at the *OsmiR528* site, due to that *EarI* based RFLP can only detect edits at the last three nucleotides of the target sequence (Figure 1C). In this case, DNA with edits at other regions of the target sequence can still be digested like unedited DNA. This data suggests that Cas12a RNPs can be efficiently delivered into rice cells, resulting in >90% editing efficiencies. Engineered AsCas12a Ultra has enhanced editing activity over AsCas12a and is on par with LbCas12a and LbCas12a-E795L. The data correlates with human cells where AsCas12a Ultra showed superior editing activity (Zhang L. et al., 2021). The reason that AsCas12a Ultra did not outperform LbCas12a and LbCas12a-E795L as in human cells (Zhang L. et al., 2021) could be because a 32°C temperature, not 37°C, was used for rice cell transfection.

### RNP Delivery Outperforms Plasmid Delivery in Rice Cells

**FIGURE 2:**
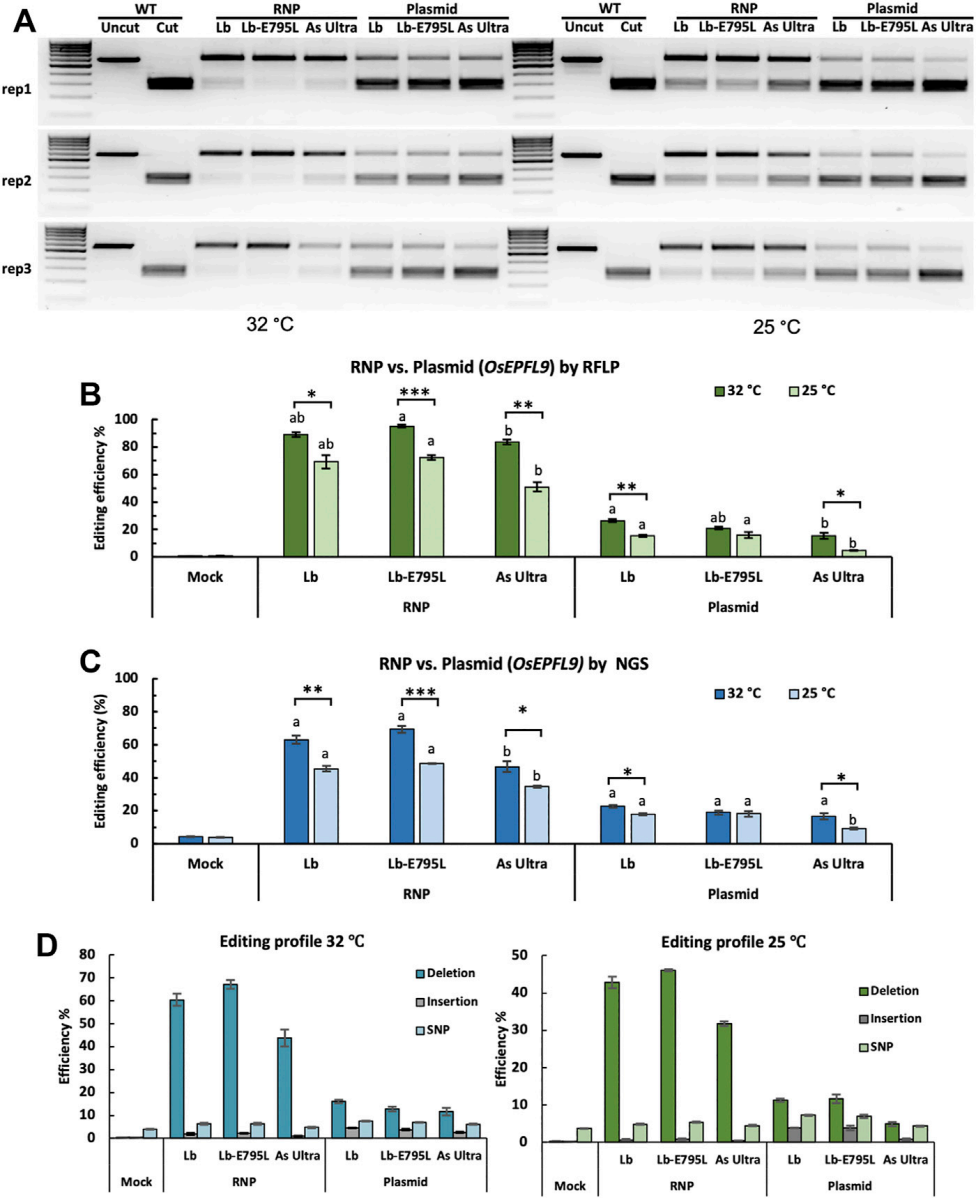
Comparison of Cas12a genome editing using RNP and plasmid delivery. Editing efficiencies of three Cas12a variants in rice cells at the *OsEPFL9* site with RNP delivery and plasmid delivery at 32°C and 25°C. **(A)** Editing at the target site is assessed by RFLP as revealed by DNA gel electrophoresis. **(B)** Quantification of editing frequencies based on the RFLP assay. **(C)** Quantification of editing frequencies by amplicon deep sequencing. **(B,C)** Editing efficiencies of three Cas12a variants using the same delivery method at the same temperature are compared using Tukey’s Honest Significant Difference (HSD) test. Treatments with the same letter are not significantly different when α = 0.05. Editing efficiencies at different temperatures are compared using Student’s t-test. Significant differences were indicated using one asterisk (*p* < 0.05), two asterisks (*p* < 0.01) or three asterisks (*p* < 0.001). **(D)** Editing profiles of three Cas12a variants in rice cells at the *OsEPFL9* site with RNP delivery and plasmid delivery at 32°C and 25°C. Data are presented as mean values ±SEM of three biologically independent replicates.

The high editing efficiency observed with RNP delivery in rice protoplasts suggests that RNP CRISPR-Cas12a delivery could result in improved editing efficiencies over traditional plasmid delivery methods. To compare editing efficiencies between RNP delivery and plasmid delivery, three of the highest performing Cas12a variants, LbCas12a, LbCas12a-E795L, and AsCas12a Ultra, were used to target the *OsEPFL9* site at two temperatures, 32°C and 25°C, which are relevant temperatures for plant genome editing and tissue culture. Both RFLP (Figures 2A,B) and next generation sequencing (NGS) of PCR amplicons (Figures 2C,D) methods were used to detect editing efficiencies. The results from these two methods were highly consistent with each other, indicating RFLP is a reliable method to detect editing efficiencies, although it is much cheaper and faster than NGS. RFLP revealed slightly higher editing efficiencies than NGS, possibly due to the different sensitivity of these two methods. RFLP assays showed significantly higher editing efficiencies were obtained using RNP delivery than plasmid delivery at both temperatures (Figures 2A–C). Notably, RNP delivery resulted in two-fold or higher editing efficiencies over plasmid delivery at both temperatures, based on the RFLP assay (Figure 2B) and deep sequencing of PCR amplicons (Figure 2C). RNP delivery of LbCas12a-E795L generated the highest editing frequency at 32°C, 69.4% as quantified by amplicon deep sequencing (Figure 2C). In all conditions except plasmid delivery of LbCas12a-E795L, the Cas12a nucleases performed better at 32°C than at 25°C (Figures 2B,C). When Cas12a nucleases and crRNAs were delivered as RNPs, LbCas12a-E795L showed significantly higher editing efficiency than AsCas12a Ultra at both temperatures (Figures 2B,C). When CRISPR reagents were delivered as plasmids, LbCas12a showed significantly higher editing efficiency than AsCas12a Ultra at 32°C, while LbCas12a and LbCas12a-E795L both showed significantly higher editing efficiency than AsCas12a Ultra at 25°C. Overall, Cas12a nucleases showed higher activity at 32°C than 25°C, except when LbCas12a-E795L was delivered as plasmids. AsCas12a Ultra is more sensitive to lower temperatures than LbCas12a and LbCas12a-E795L. These results are consistent with our previous observation on Cas12a temperature sensitivity and hyper temperature sensitivity of AsCas12a in plants (Malzahn et al., 2019). Notably, RNP delivery even at the lower temperature (25°C) outperformed plasmid delivery at the higher temperature (32°C). The data hence support that RNP delivery of CRISPR-Cas12a can drastically improve editing efficiencies at low temperatures.

We further analyzed the editing profiles using deep sequencing data (Figure 2D). The majority of the edits were deletions, as reported previously (Tang et al., 2017). No obvious differences were observed among the Cas12a variants. Similar editing patterns were obtained using different delivery methods at both temperatures, supporting that Cas12a nuclease activity, not DNA repair, and is impacted by temperature (Malzahn et al., 2019).

### RNP Efficiency is Dosage-Dependent

**FIGURE 3:**
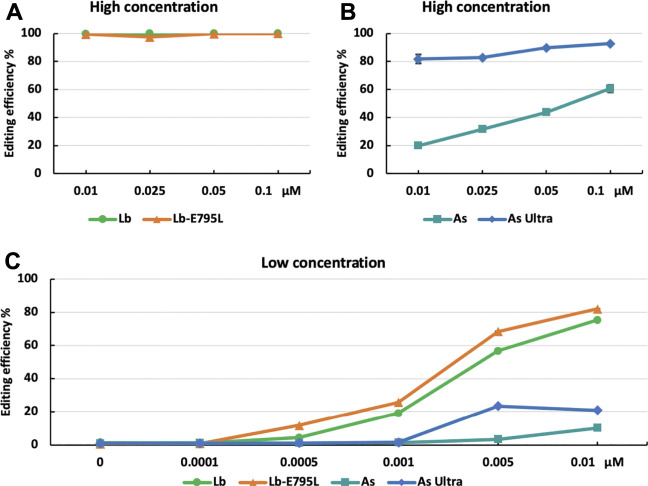
Cas12a editing efficiencies at different dosages in rice cells with RNP delivery. Editing efficiencies of four Cas12a variants were tested in rice cells with RNP delivery at the *OsEPFL9* site at 32°C. Editing efficiencies are calculated using the RFLP assay with high RNP concentrations **(A,B)** and low RNP concentrations **(C)**. Data are presented as mean values ±SEM of three biologically independent replicates.

The high editing efficiencies observed with RNP delivery of CRISPR-Cas12a suggest that the RNP dosage used could be near saturation. To investigate the effects of dosage on genome editing using RNP delivery, we edited the *OsEPFL9* site using different concentrations of RNPs. At high concentrations (0.01, 0.025, 0.05, and 0.1 µM), LbCas12a and LbCas12a-E795L maintained approximately 100% editing efficiency assessed by RFLP (Figure 3A). At 0.01 µM, AsCas12a Ultra dropped to 81.8%, while AsCas12a dropped to 19.9% (Figure 3B). These results indicate that AsCas12a Ultra is more efficient than AsCas12a in rice cells, as observed in human cells (Zhang L. et al., 2021). At low concentrations (0.0001, 0.0005, 0.001, and 0.005 µM), editing efficiencies of LbCas12a and LbCas12a-E795L dropped along with the dosage titration (Figure 3C). AsCas12a Ultra and AsCas12a showed much lower activity than at high concentrations (Figure 3C). The comparison at low RNP concentrations revealed that LbCas12a-E795 is slightly more robust than LbCas12a (Figure 3C). These results suggest that 0.01 µM RNPs of LbCas12a or LbCas12a-E795L are sufficient to obtain highly efficient genome editing in rice cells.

### A 1:1 Cas12a:crRNA Ratio is Sufficient for Efficient Genome Editing in Rice Cells

**FIGURE 4:**
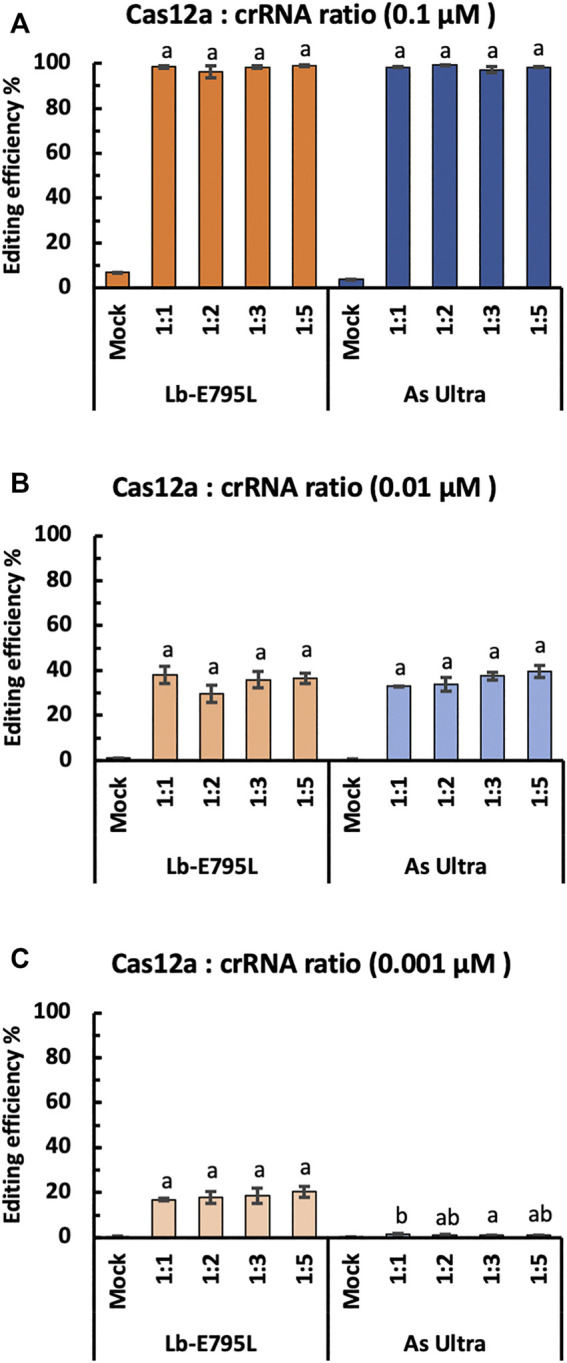
Cas12a editing efficiencies at different Cas12a:crRNA ratios in rice cells with RNP delivery. Editing efficiencies of two Cas12a variants were tested in rice cells with RNP delivery at the *OsEPFL9* site at 32°C. Editing efficiencies are calculated using the RFLP assay with three RNP concentrations, 0.1 µM **(A)**, 0.01 µM **(B)** and 0.001 µM **(C)**. Data are presented as mean values ±SEM of three biologically independent replicates. Editing efficiencies using four different Cas12a:crRNA ratios are compared using Tukey’s Honest Significant Difference (HSD) test. Treatments with the same letter are not significantly different when α = 0.05.

For RNP delivery, it is important to know the optimal molar ratio of Cas12a:crRNA. Theoretically, a 1:1 ratio is expected to configure the RNP complex with minimal waste of either component. However, more crRNA is often used in practice, due to concerns associated with crRNA degradation (Zhang et al., 2021b). To investigate the effects of Cas12a:crRNA ratio on genome editing using RNP delivery, we edited the *OsEPFL9* site using four Cas12a:crRNA ratios, 1:1, 1:2, 1:3, and 1:5, at RNP concentrations of 0.1, 0.01 and 0.001 µM. We compared LbCas12a-E795 and AsCas12a Ultra in this experiment and assessed editing efficiency by RFLP assay. Regardless of RNP dosage, no significant differences were observed when using different Cas12a:crRNA ratios for LbCas12a-E795L and AsCas12a Ultra (Figure 4). Interestingly, sequential reductions of RNP concentration results in ∼50% reductions of editing frequencies in each concentration (Figure 4). At 0.001 µM, editing activity by AsCas12a Ultra was undetectable regardless of Cas12a:crRNA ratios (Figure 4C), which is consistent with the earlier data (Figure 3C). Our results suggest that the 1:1 Cas12a:crRNA ratio is optimal for efficient genome editing in rice cells.

### crRNA Modification With RNP has Minimal Effects on Genome Editing Efficiency

**FIGURE 5:**
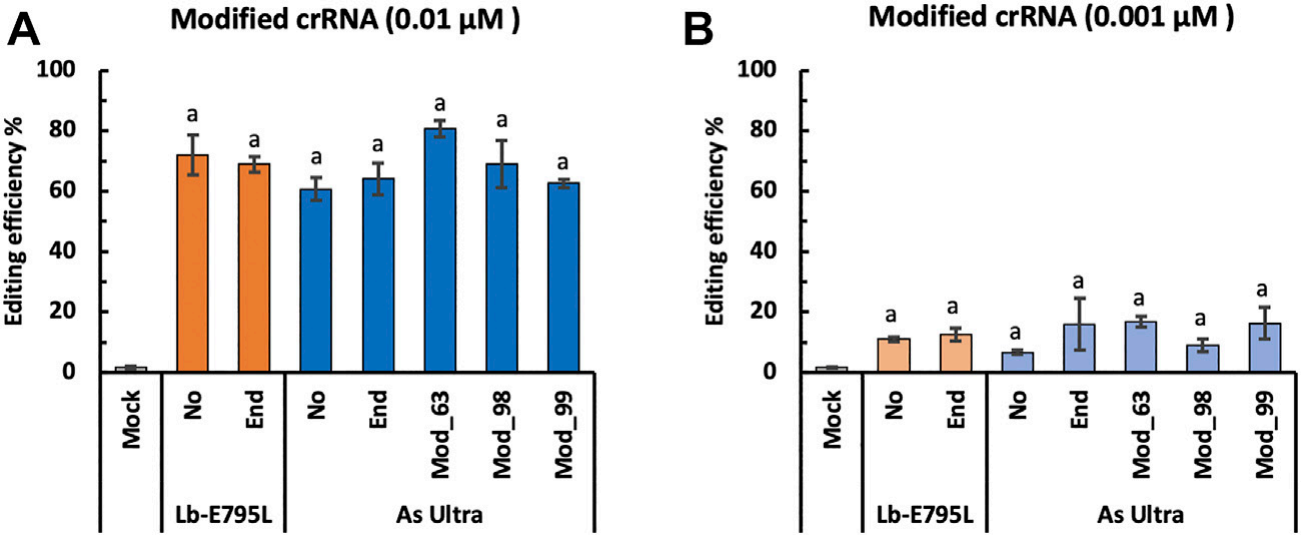
Cas12a editing efficiencies using modified crRNA in rice cells with RNP delivery. Editing efficiencies of two Cas12a variants were tested in rice cells with RNP delivery at the *OsEPFL9* site at 32°C. Editing efficiencies are calculated using the RFLP assay with two RNP concentrations, 0.01 µM **(A)** and 0.001 µM **(B)**. Data are presented as mean values ±SEM of three biologically independent replicates. Editing efficiencies using unmodified crRNAs or crRNAs with different types of modifications are compared using Tukey’s Honest Significant Difference (HSD) test. Treatments with the same letter are not significantly different when α = 0.05.

RNA modification could potentially enhance their stability in cells (Hendel et al., 2015). We tested one type of crRNA modification for LbCas12a-E795L and four types of crRNA modification for AsCas12a Ultra at the *OsEPFL9* site, using RNP concentrations of 0.01 and 0.001 µM. All of these modifications will presumably protect crRNAs from degradation, before and after transfection. Strikingly, no significant differences were observed with or without end modification, or between different types of modification at either RNP concentration (Figure 5). These results suggest that crRNA modifications have minimal effects on genome editing efficiency in rice cells. We speculate that the RNP assembly is so efficient that crRNAs are minimally exposed to RNases before and after transfection. On the contrary, DNA delivery of CRISRP-Cas12a requires individual expression of Cas12a and crRNA before they form RNPs within cells, thereby providing many opportunities for crRNA degradation. Hence, our data support that RNPs of CRISPR-Cas12a are highly stable in rice cells.

### A Nuclear Localization Signal (NLS) Is Critical for RNP Delivery

**FIGURE 6:**
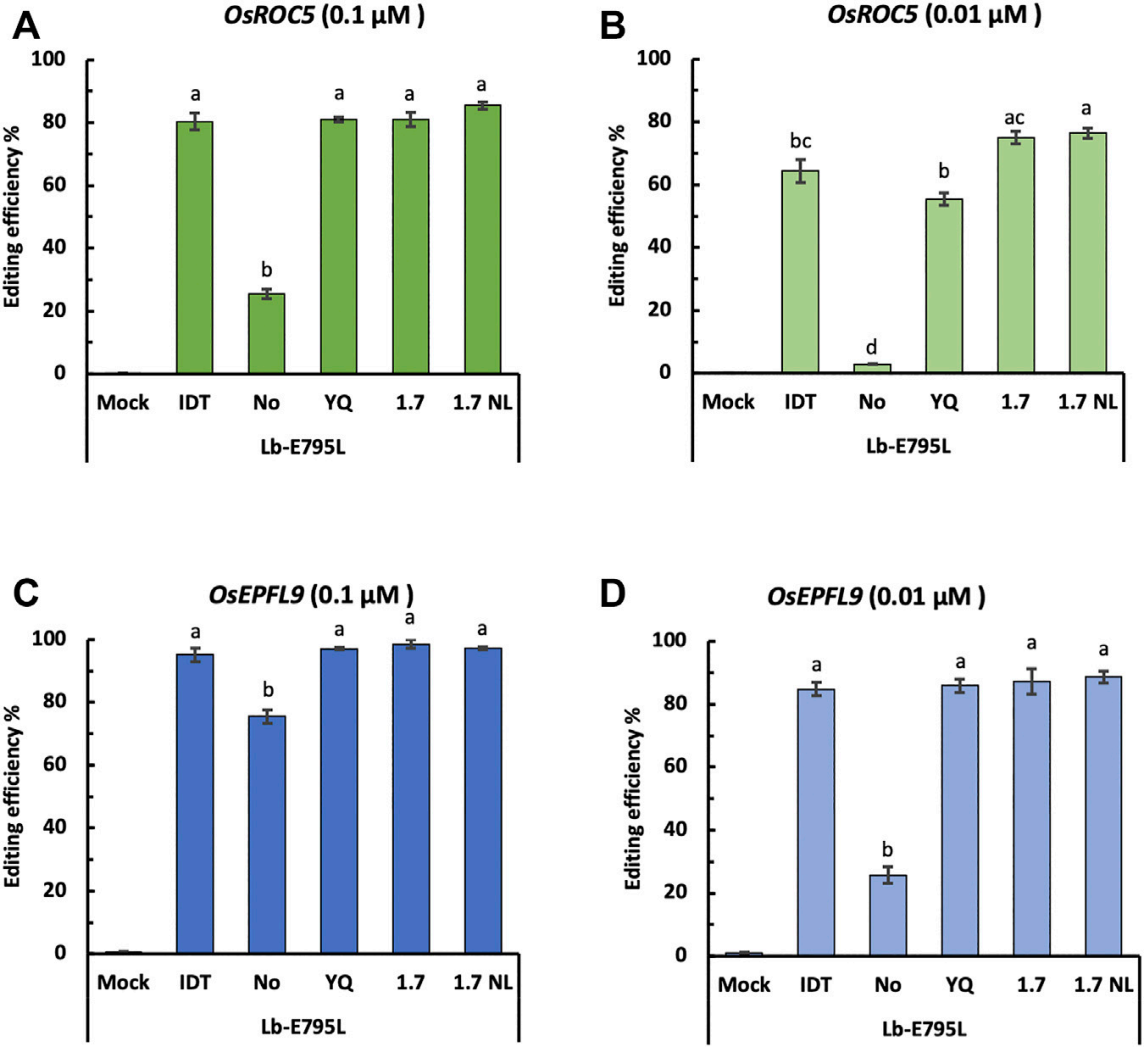
Cas12a editing efficiencies using different nuclear localization signals (NLSs) in rice cells with RNP delivery. Editing efficiencies of Cas12a-E795L without or with different NLSs were tested in rice cells with RNP delivery at the two target sites at 32°C. Editing efficiencies are calculated using the RFLP assay with two RNP concentrations, 0.01 and 0.001 µM, at the *OsROC5* **(A,B)** and *OsEPFL9* **(C,D)** sites. Data are presented as mean values ±SEM of three biologically independent replicates. Editing efficiencies using Cas12a nucleases without or with different types of NLSs are compared using Tukey’s Honest Significant Difference (HSD) test. Treatments with the same letter are not significantly different when α = 0.05.

NLS is important for Cas12a to enter the nucleus for genome editing. It is possible CRISPR-Cas12a genome editing efficiency may be improved through NLS optimization. To this end, we tested four types of NLSs (Supplementary Table S4) for LbCas12a-E795L at the *OsEPFL9* and *OsROC5* sites with RNP concentrations of 0.1 and 0.01 µM. No significant differences for editing efficiency were observed using different types of NLSs as measured by RFLP, except at the *OsROC5* site with 0.01 µM RNPs (Figure 6). Without NLS, editing efficiencies at the four target sites ranged from none (at *OsROC5*) (Figure 6B) to 75.4% (at *OsEPFL9*) (Figure 6C). The relatively high editing efficiency by LbCas12a-E795L at *OsEPFL9* even without NLS is likely attributed to the high efficiency of the crRNA for this site and the high RNP concentration (0.1 µM) (Figure 6C). At 0.01 µM of RNPs, the editing activity by LbCas12a-E795L significantly dropped without an NLS at this same target site (Figure 6D). These data suggest that nuclear entry is a limiting step for Cas12a:crRNA RNPs and NLS is critical for efficient Cas12a mediated genome editing.

### Cas12a Genome Editing in Citrus Cells With RNP Delivery

**FIGURE 7:**
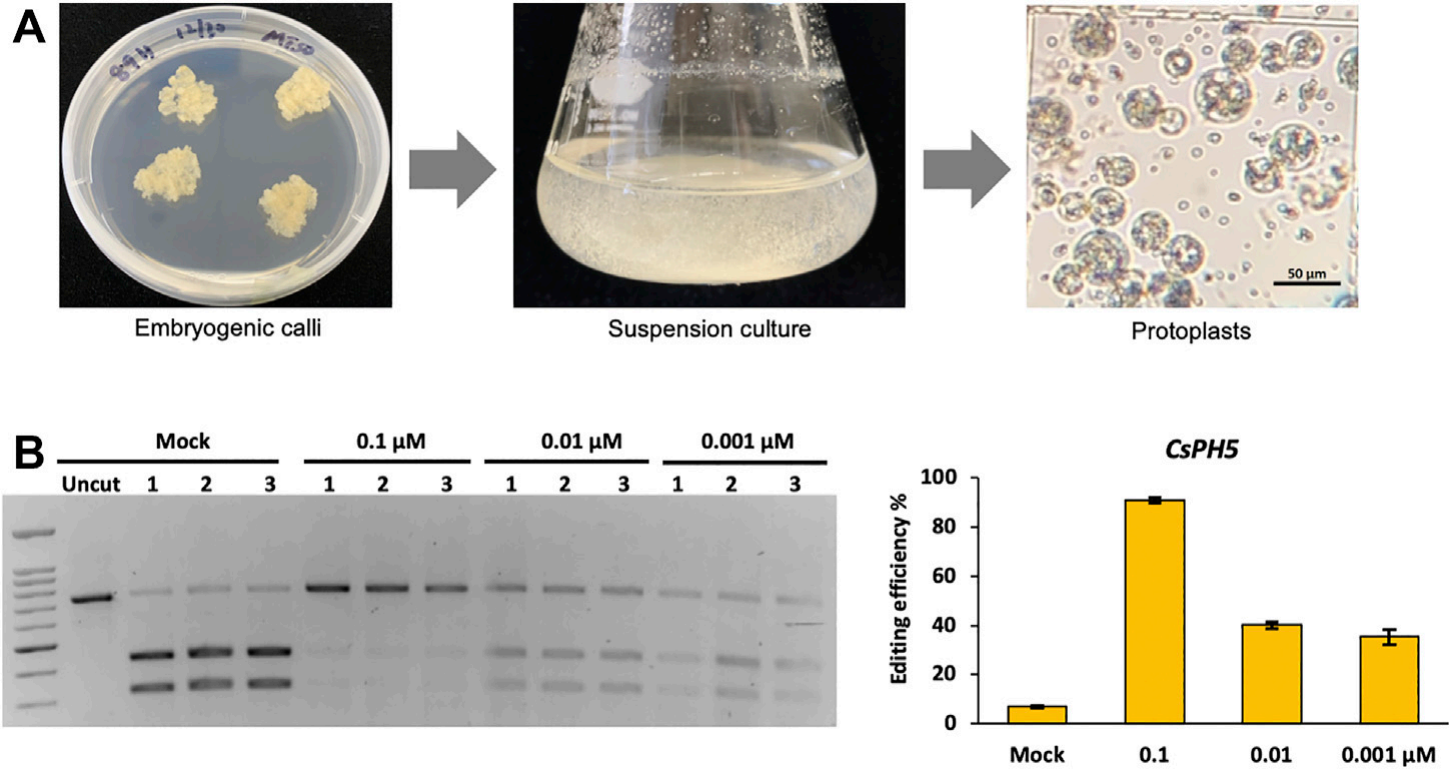
LbCas12a genome editing in citrus cells with RNP delivery. **(A)** A pipeline for production of protoplasts from an embryogenic sweet orange line, Hamlin 89. **(B)** Editing efficiencies of LbCas12a are calculated using the RFLP assay with three RNP concentrations, 0.1, 0.01, and 0.001 μM at gene *CsPH5* at 28°C. The left panel shows the RFLP assay, and the right panel shows the qualification of mutation frequencies based on the RFLP assay. Data are presented as mean values ±SEM of three biologically independent replicates.

Although our initial RNP CRISPR-Cas12a delivery data was derived from rice protoplasts, the protocols developed in principle should be widely applicable to protoplasts systems derived from other plant species. To expand the application of Cas12a-mediated genome editing using RNP delivery, we also conducted genome editing using LbCas12a in the protoplasts derived from embryogenic calli of Hamlin sweet orange, a dicot fruit tree species. We first established a pipeline that yielded high quality protoplasts from suspension culture cells derived from an embryogenic sweet orange callus line Hamlin 89 (Figure 7A). With these protoplasts, three RNP concentrations, 0.1, 0.01, and 0.001 µM, were used to assess genome editing at the *CsPH5* gene. The RFLP assay showed 90.8% editing efficiency at 0.1 µM, 40.1% editing efficiency at 0.01 µM, and 35.3% editing efficiency at 0.001 µM (Figure 7B). Although these editing efficiencies were slightly overestimated due to residual uncut bands by RFLP in the mock samples, the results clearly indicate that RNP Cas12a delivery provides as a robust means for highly efficient genome editing in citrus cells. Notably, this experiment was conducted at 28°C. Whether this genome editing efficiency in citrus can be reproduced or improved at other temperatures needs to be further investigated. By contrast, our attempts of plasmid delivery of CRISPR-Cas12a yielded nearly undetectable editing activity in citrus protoplasts (data not shown). Since the protoplasts used were derived from embryogenic calli of citrus that have high potential for plant regeneration, high efficiency genome editing by RNP delivery of CRISPR-Cas12a suggests a promising avenue to engineering transgene-free genome-edited citrus varieties that are potentially resistant to devastating diseases such as citrus greening disease. A protocol that renders successful regeneration from such protoplasts to embryogenic calli and subsequently to whole plants will need to be established. It is also envisioned that absence of selection as a nature of RNP delivery should not be a problem due to the high editing efficiency.

## Conclusion

In this study, we comprehensively investigated the effects of different Cas12a nucleases, temperature, dosage, Cas12a:crRNA ratio, crRNA modification, and NLS on genome editing efficiency in protoplasts. We demonstrate highly efficient genome editing using Cas12a delivered as RNPs in rice and citrus protoplasts. High efficiency genome editing based on RNP CRISPR-Cas12a delivery in plant protoplasts demonstrated in this study is consistent with recent report of RNP delivery of CRISPR-Cas12a using biolistic delivery in rice (Banakar et al., 2020) and maize (Dong et al., 2021). We note that editing efficiencies may vary in different experiments for the same RNP formulation especially when lower concentrations of RNPs are used, which may be attributed to RNP batch variations and/or repeated freeze-thaw cycles during usage. For achieving more consistent results, we recommend preparing the fresh reagents in small aliquots for storage and conducting the experiments at the same time for any comparisons. Nevertheless, this study provides an efficient platform and informative guidelines for Cas12a-mediated genome editing using RNP delivery in plant cells. The system is suitable for varying applications such as transient testing of Cas12a nucleases, screening crRNAs, and testing novel CRISPR tools. Further, it can be used to generate transgene-free genome edited plants from protoplasts. Plant regeneration from protoplasts is currently non-trivial for most plant species (Zhang et al., 2021b) and is a major bottleneck for successful generation of Cas12a genome-edited plants via highly efficient and transgene-free RNP delivery into protoplasts.

## Data Availability

The original contributions presented in the study are included in the article/Supplementary Material, Next-generation sequencing (NGS) data are available in the National Center for Biotechnology information (NCBI) database under Sequence Read Archive (SRA) BioProject #PRJNA789279.
