# Supplementary material for: Highly Efficient Genome Editing in Plant Protoplasts by Ribonucleoprotein Delivery of CRISPR-Cas12a Nucleases
Source: Front Genome Ed. 2022 Jan 31;4:780238. doi: 10.3389/fgeed.2022.780238 (PMC8842731; doi:10.3389/fgeed.2022.780238)
Supplement: Supplementary file 1 [file Table1.DOCX]

**Supplementary Table 1.** **Target sites, crRNAs and enzymes for RFLP used in this study**

| **Target site** | **crRNA sequence with direct repeat** | **Enzyme for RFLP assay** |
| --- | --- | --- |
| *OsPDS* | Lb: TAATTTCTACTAAGTGTAGATGAGTGAAATCTCTTGTCTTAAGG | AflII |
|  | As: TAATTTCTACTCTTGTAGATGAGTGAAATCTCTTGTCTTAAGG |  |
| *OsROC5* | Lb: TAATTTCTACTAAGTGTAGATTGCTTCCTGCAATGCCGGTAGAC | AccI |
|  | As: TAATTTCTACTCTTGTAGATTGCTTCCTGCAATGCCGGTAGAC |  |
| *OsmiR528* | Lb: TAATTTCTACTAAGTGTAGATCCTCTCTCTCCTGTGCTTGCCTC | EarI |
|  | As: TAATTTCTACTCTTGTAGATCCTCTCTCTCCTGTGCTTGCCTC |  |
| *OsEPFL9* | Lb: TAATTTCTACTAAGTGTAGATAAGAAGGGTTATGGCCAATGCTT | MscI |
|  | As: TAATTTCTACTCTTGTAGATAAGAAGGGTTATGGCCAATGCTT |  |
| *CsPH5* | Lb: TAATTTCTACTAAGTGTAGATGCCCCAACAAGCTAGAAGAGAAA | EarI |

**Supplementary Table 2.** **Oligonucleotides used in this study**

| **Name** | **Sequence** | **Usage** |
| --- | --- | --- |
| TTTN-OsEPFL9-crRNA1-F | TAGATAAGAAGGGTTATGGCCAATGCTT | crRNA cloning |
| TTTN-OsEPFL9-crRNA1-R | GGCCAAGCATTGGCCATAACCCTTCTTA | crRNA cloning |
| OsPDS-F | CTGGCTGCCTGTCATCTATGAA | PCR primer for RFLP assay |
| OsPDS-R | CCAAAACATCCCTTGCCTCA | PCR primer for RFLP assay |
| OsROC5-F | CTTATGTTCCGTTCCAATCCT | PCR primer for RFLP assay |
| OsROC5-R | CCTACACTTCACATTTCCACCT | PCR primer for RFLP assay |
| OsmiR528-F | AACCCTCTGAGTCACGAGTC | PCR primer for RFLP assay |
| OsmiR528-R | GCAAAAAAGAGCTAGCCCAC | PCR primer for RFLP assay |
| OsEPFL9-F | GAGATGAGCCATCTGGTGGT | PCR primer for RFLP assay |
| OsEPFL9-R | CCTTGATGTTCTCCTGCAAA | PCR primer for RFLP assay |
| CsPH5-F | AACCCTCTGAGTCACGAGTC | PCR primer for RFLP assay |
| CsPH5-R | GCAAAAAAGAGCTAGCCCAC | PCR primer for RFLP assay |
| OsEPFL9-HTS-F | CTCCCCTTGTGTTGCTTCTC | PCR primer for amplicon deep sequencing |
| OsEPFL9-HTS-R | GCAACGAGCATGACTGAAGA | PCR primer for amplicon deep sequencing |

**Supplementary Table 3.** **Cas12a nucleuses from IDT used in this study**

| **Cas12a nucleases** | **Cas12a protein sequence** | **Linker** | **NLS** |
| --- | --- | --- | --- |
| LbCas12a | Wild type | N/A | 2X Original (SV40) |
| LbCas12a-E795L ([Zhang et al., 2021a](#_ENREF_43)) | Optimized mutant (E795L) | Improved | Improved |
| AsCas12a ([Zhang et al., 2021a](#_ENREF_43)) | Wild type | Improved | Optimized  (OpT) |
| AsCas12a Ultra ([Zhang et al., 2021a](#_ENREF_43)) | Optimized mutant (M537R, F870L) | Improved | Improved |

**Supplementary Table 4.** **Different crRNA modifications compared in this study**

| crRNA modification | Annotation  r = RNA bases, m = 2’O-met bases, * = Phosphorothioate bonds |
| --- | --- |
| End | /AltR1/rUrArArUrUrUrCrUrArCrUrCrUrUrGrUrArGrArUrGrArGrUrGrArArArUrCrUrCrUrUrGrUrCrUrUrArArGrG/AltR2/ |
| Mod_63 | mU*rA*rA*rUmUmUmCmUmAmCrUmCmUmUrGmUmAmGmArUrGrArGrUrGrArArArUrCrUrCrUrUrGrUrCrUrUrA*rA*rG*rG |
| Mod_98 | mUrArArUmUmUmCmUmAmCrUmCmUmUrGmUmAmGmArUrGrArGrUrGrArArArUrCrUrCrUrUrGrUrCrUrUrArArGrG |
| Mod_99 | mU*rA*rArUrUrUrCrUrArCrUrCrUrUrGrUrArGrArUrGrArGrUrGrArArArUrCrUrCrUrUrGrUrCrUrUrA*mA*mG*mG |
